# Supplementary material for: Integrated Metabolomics and Lipidomics Analysis Reveals Lipid Metabolic Disorder in NCM460 Cells Caused by Aflatoxin B1 and Aflatoxin M1 Alone and in Combination
Source: Toxins (Basel). 2023 Mar 31;15(4):255. doi: 10.3390/toxins15040255 (PMC10146203; doi:10.3390/toxins15040255)
Supplement: Supplementary file 1 [file toxins-15-00255-s001.zip › toxins-2256265-supplementary.pdf]

toxins

MDPI

Supplementary Materials: Integrated Metabolomics and Lipidomics Analysis Reveals Lipid Metabolic Disorder in NCM460 Cells Caused by Aflatoxin B1 and Aflatoxin M1 Alone and in Combination

Xue Yang, Xue Li, Yanan Gao, Jiaqi Wang and Nan Zheng

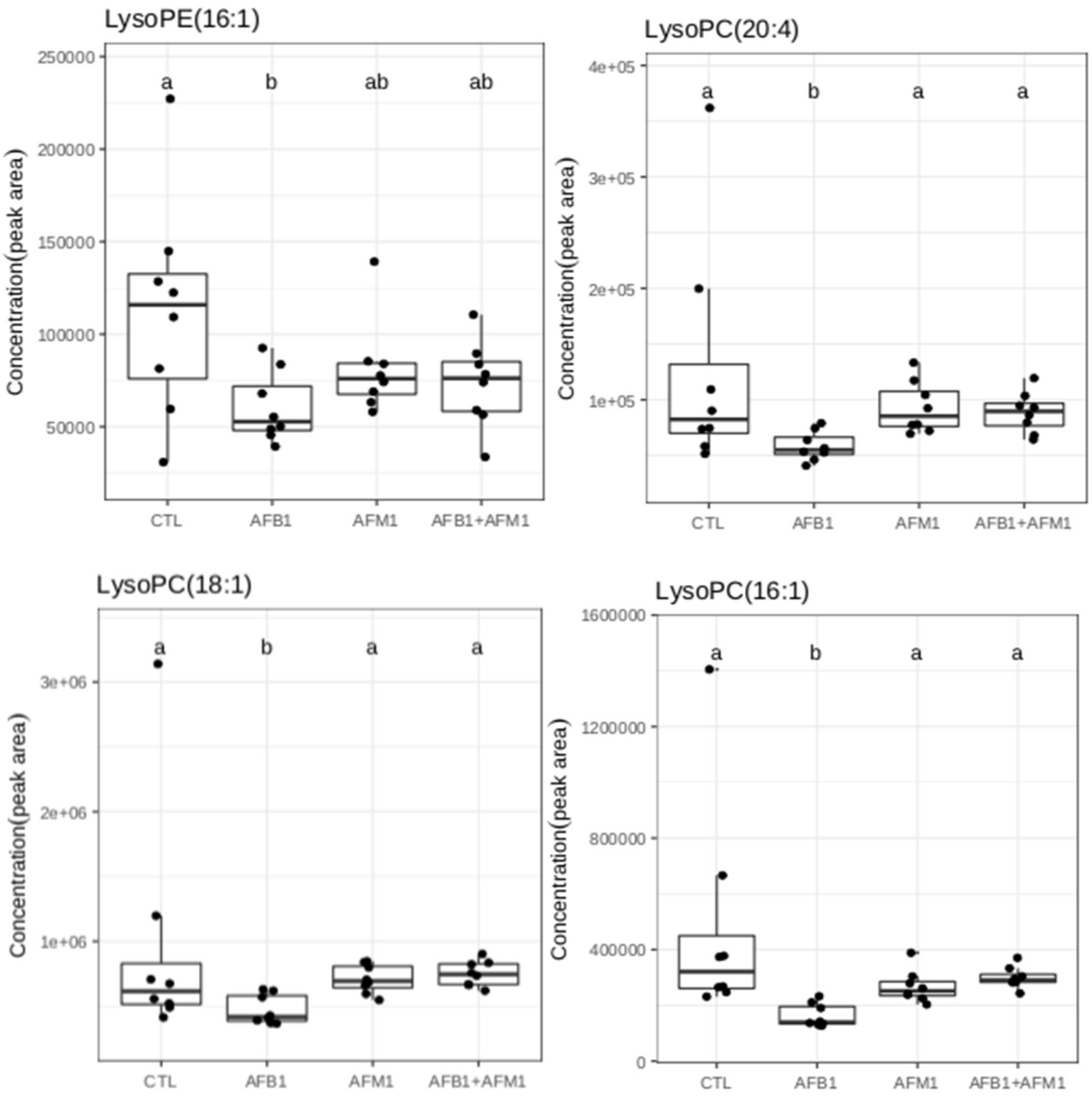

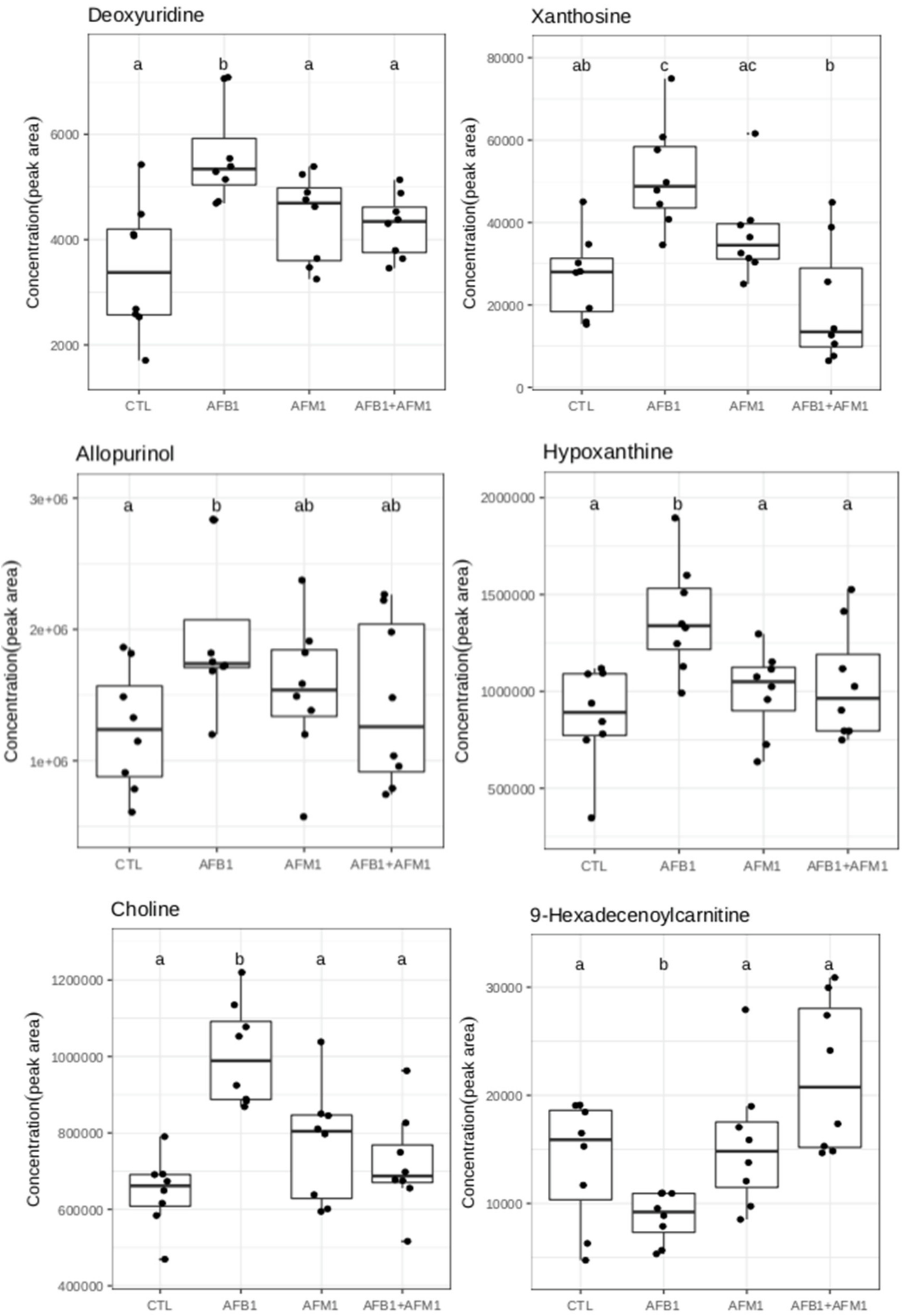
Deoxyuridine
a b a a
2000 4000 6000
Concentration(peak area)
CTL AFB1 AFM1 AFB1+AFM1
Xanthosine
ab c ac b
0 20000 40000 60000 80000
Concentration(peak area)
CTL AFB1 AFM1 AFB1+AFM1
Allopurinol
a b ab ab
1e+06 2e+06 3e+06
Concentration(peak area)
CTL AFB1 AFM1 AFB1+AFM1
Hypoxanthine
a b a a
500000 1000000 1500000 2000000
Concentration(peak area)
CTL AFB1 AFM1 AFB1+AFM1
Choline
a b a a
400000 600000 800000 1000000 1200000
Concentration(peak area)
CTL AFB1 AFM1 AFB1+AFM1
9-Hexadecenoylcarnitine
a b a a
10000 20000 30000
Concentration(peak area)
CTL AFB1 AFM1 AFB1+AFM1

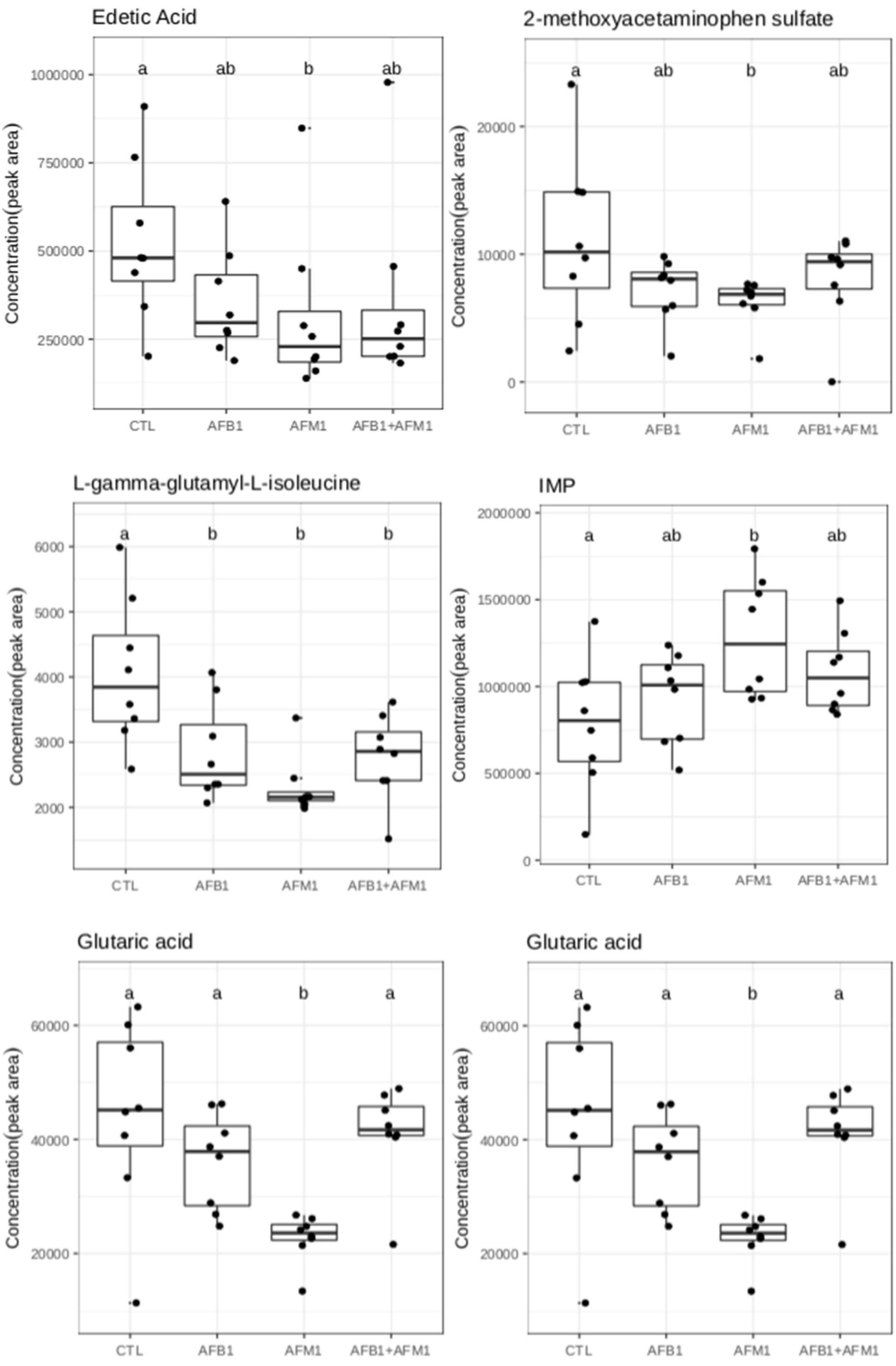

Edetic Acid
a
ab
b
ab
1000000
750000
500000
250000
Concentration(peak area)
CTL
AFB1
AFM1
AFB1+AFM1
2-methoxyacetaminophen sulfate
a
ab
b
ab
20000
10000
0
Concentration(peak area)
CTL
AFB1
AFM1
AFB1+AFM1
L-gamma-glutamyl-L-isoleucine
a
b
b
b
6000
5000
4000
3000
2000
Concentration(peak area)
CTL
AFB1
AFM1
AFB1+AFM1
IMP
a
ab
b
ab
2000000
1500000
1000000
500000
0
Concentration(peak area)
CTL
AFB1
AFM1
AFB1+AFM1
Glutaric acid
a
a
b
a
60000
40000
20000
Concentration(peak area)
CTL
AFB1
AFM1
AFB1+AFM1
Glutaric acid
a
a
b
a
60000
40000
20000
Concentration(peak area)
CTL
AFB1
AFM1
AFB1+AFM1

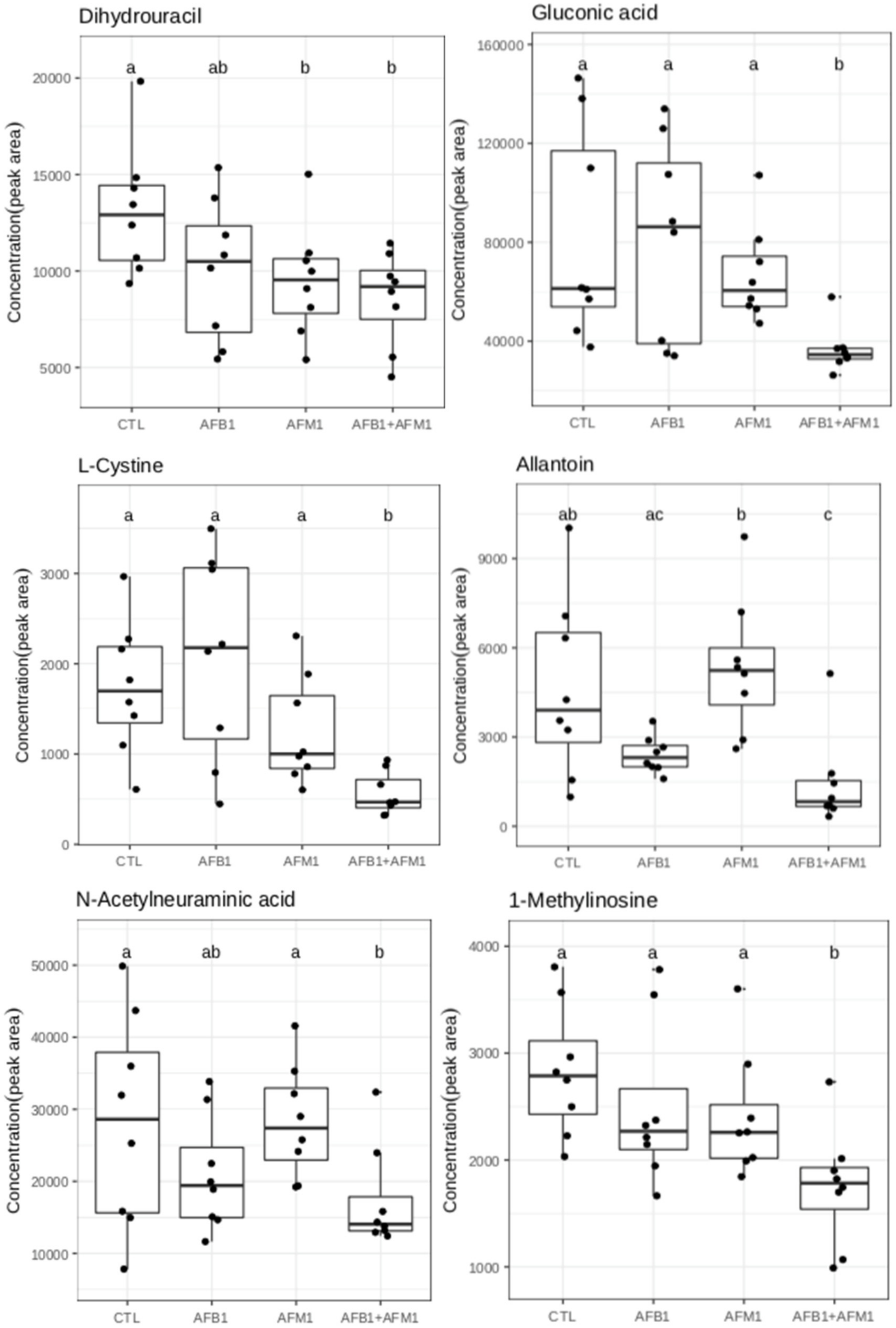

Dihydrouracil
Gluconic acid
L-Cystine
Allantoin
N-Acetylneuraminic acid
1-Methylinosine
Concentration(peak area)
CTL
AFB1
AFM1
AFB1+AFM1
a
ab
b
ac
c

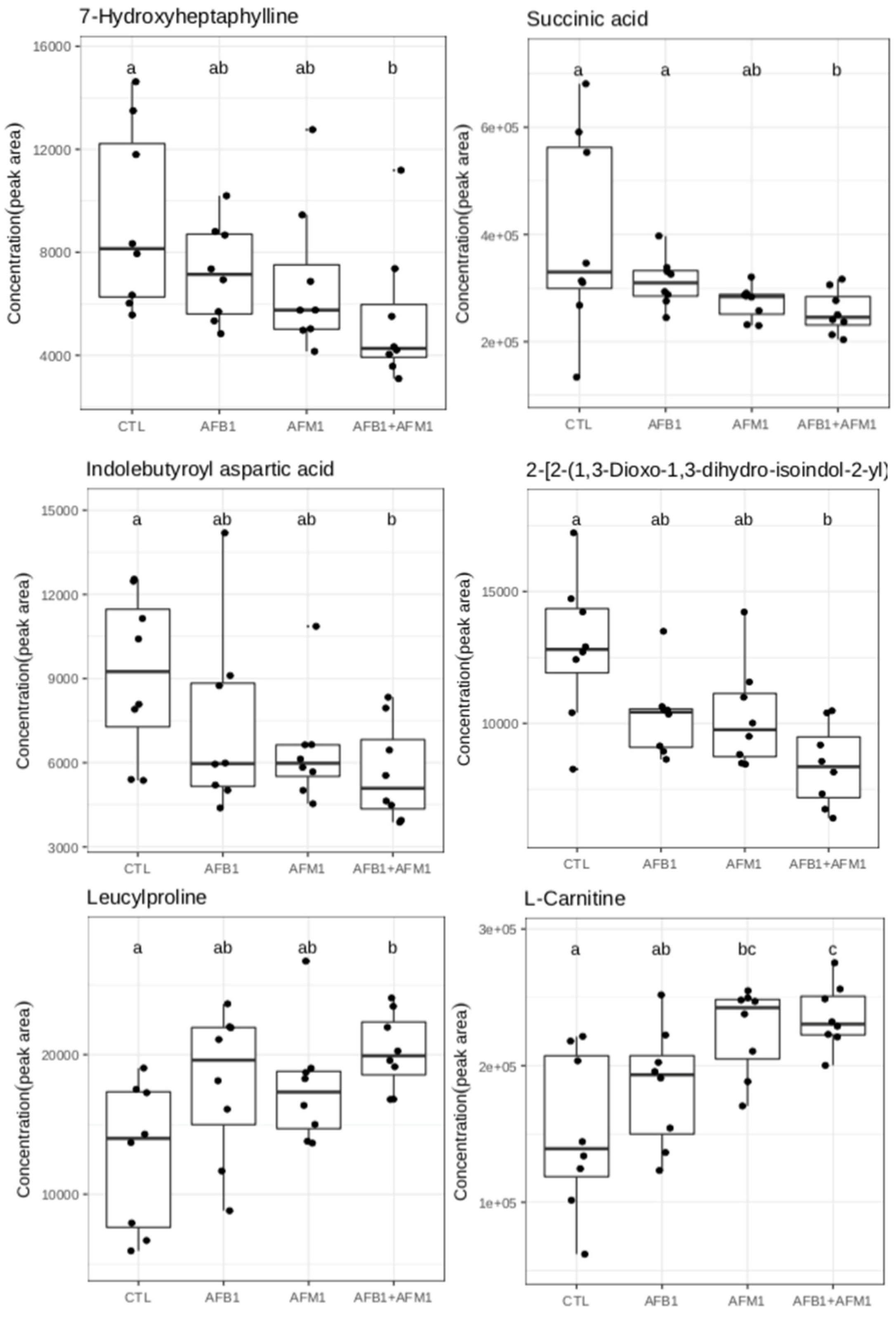
7-Hydroxyheptaphylline
16000
12000
8000
4000
a
ab
ab
b
Concentration(peak area)
CTL
AFB1
AFM1
AFB1+AFM1
Succinic acid
6e+05
4e+05
2e+05
a
a
ab
b
Concentration(peak area)
CTL
AFB1
AFM1
AFB1+AFM1
Indolebutyroyl aspartic acid
15000
12000
9000
6000
3000
a
ab
ab
b
Concentration(peak area)
CTL
AFB1
AFM1
AFB1+AFM1
2-[2-(1,3-Dioxo-1,3-dihydro-isoindol-2-yl)
15000
10000
a
ab
ab
b
Concentration(peak area)
CTL
AFB1
AFM1
AFB1+AFM1
Leucylproline
20000
10000
a
ab
ab
b
Concentration(peak area)
CTL
AFB1
AFM1
AFB1+AFM1
L-Carnitine
3e+05
2e+05
1e+05
a
ab
bc
c
Concentration(peak area)
CTL
AFB1
AFM1
AFB1+AFM1

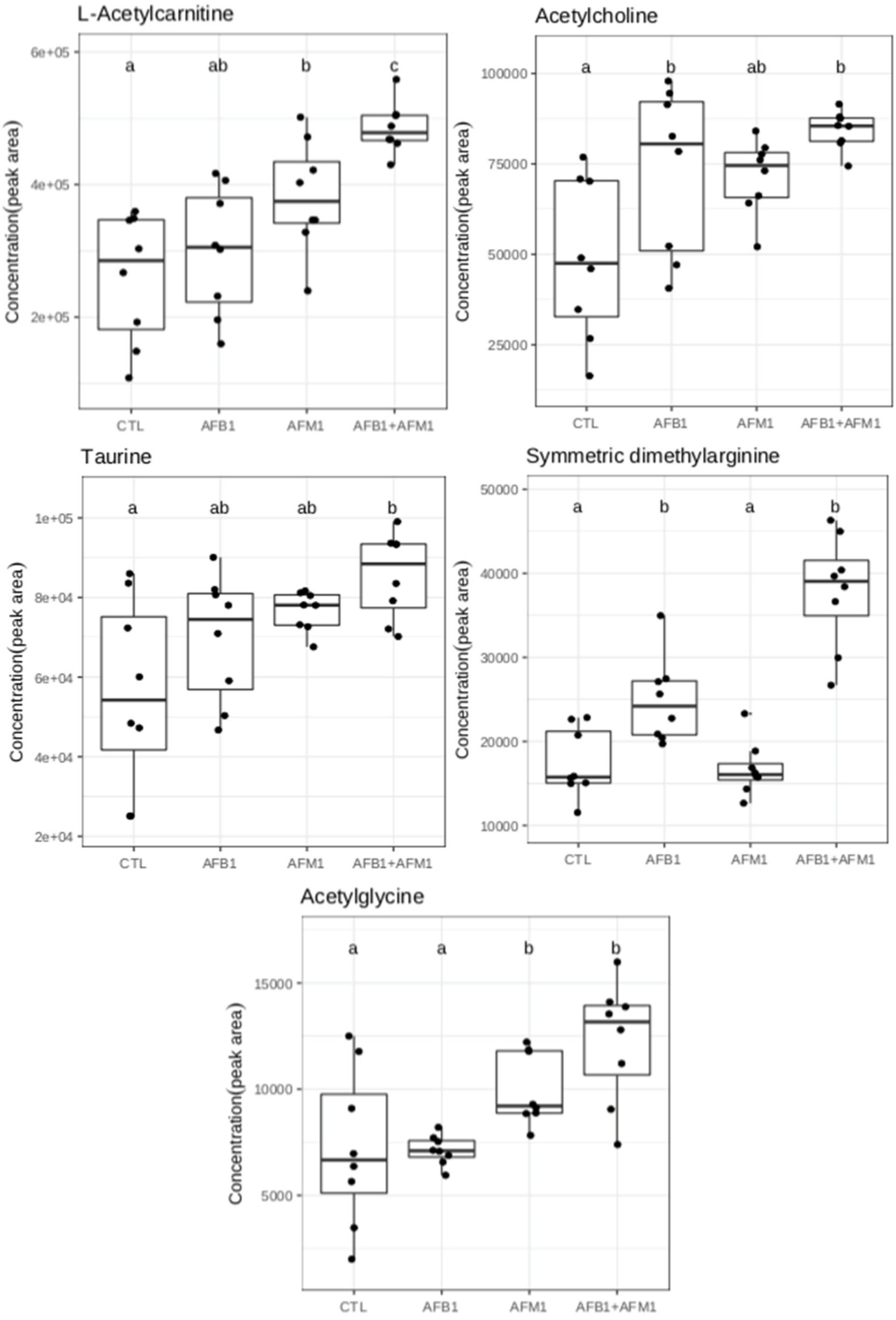

Figure S1. Boxplot further exhibited the level of each specific metabolites in different groups. Different letters (a-c) indicate significant differences ($p < 0.05$).

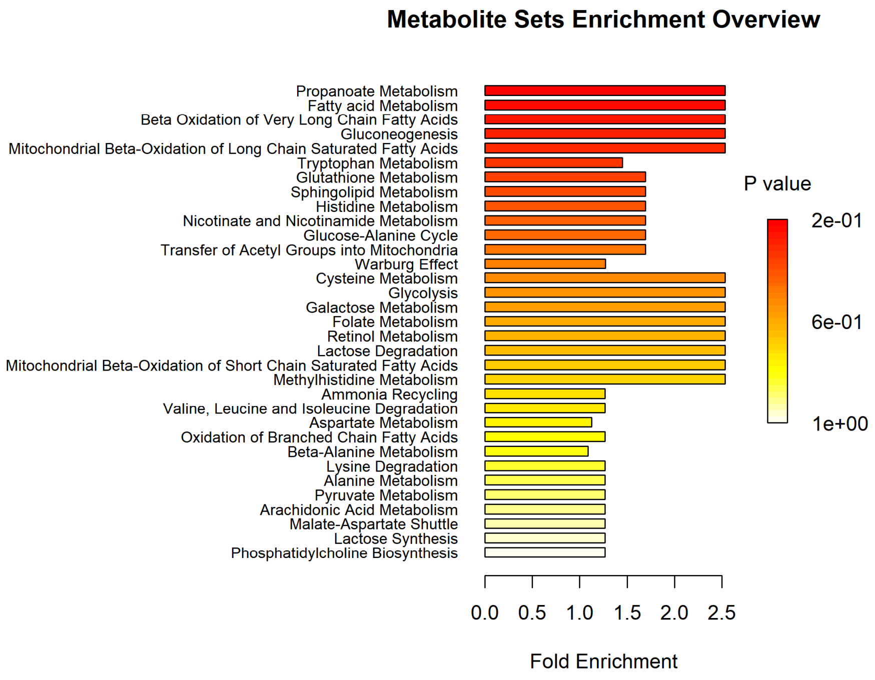

Figure S2. The metabolites sets enrichment analysis of differential expression metabolites in AFM1/CTL.
